# The impact of corporate social responsibility on firm reputation and organizational citizenship behavior: The mediation of organic organizational cultures

**DOI:** 10.3389/fpsyg.2022.1100448

**Published:** 2023-01-23

**Authors:** Hussain Ali, Jianhua Yin, Faiza Manzoor, Mengmeng An

**Affiliations:** ^1^Business School, University of International Business and Economics, Beijing, China; ^2^Department of Agricultural Economics and Management, School of Public Affairs, Zhejiang University, Hangzhou, China; ^3^School of Government, University of International Business and Economics, Beijing, China

**Keywords:** CSR, organic organizational cultures, firm reputation, employees OCB, Pakistan

## Abstract

This study investigates the impact of corporate social responsibility (CSR) on firm reputation and organizational citizenship behavior, along with the mediating inclusion of organic organizational cultures (Clan and Adhocracy) in the medium and large enterprises of Pakistan. To do the path analysis and to investigate the mediating role of organic organization culture, Smart PLS was used. For data collection, the convenience sampling technique was used and responses from 360 questionnaires were the main data source. The results displayed that CSR has a significant and optimistic effect on a firm reputation and employees’ organizational citizenship behavior (OCB). Secondly, the outcomes showed a positive and full mediation effect of organic organizational cultures between CSR and firm reputation but a partial mediation effect of organic organizational cultures between CSR and employees OCB. Further, the results demonstrated a positive and significant influence of employees’ OCB on a firm reputation. The extensive analysis of all factors of the study was autonomously examined to identify the insights that verify how the inclusion of organic organizational cultures can boost the firm reputation and employees’ OCB. Theoretical implications and future research direction are discussed.

## 1. Introduction

In an increasingly globalized business domain, intangible assets like firm reputation and worker trust are crucial. Indeed, intangible assets are critical in determining a business’s long-term sustainability and success. A firm’s reputation (FR) is stated as an overall impression that exposes the collective perception of stakeholder units (Sallah and Caesar, 2020). In recent years, academics have assessed corporate social responsibility (CSR) in multiple contexts. Increased interest in CSR sparked from the increasing globalization and international trade, which resulted in more complex business operations and triggered demand-based transparency and corporate citizenship. Academics have shown a strong interest in studying CSR in recent years, with the corporate sector providing widely the early impetus for research into CSR in the aftermath of significant scandals like WorldCom and Enron. Particularly, many studies have focused on strengthening business leadership characteristics that encourage organizations to engage in ethical and socially responsible behavior (Pasricha et al., 2018). The efficiency of social enterprises is based on meeting the expectancies of various stakeholders (Mikołajczak, 2020), and henceforth the occurrence of CSR strategy in these organizations is a subject matter of additional study (Yan et al., 2022). Theoretically, research specialists continue to investigate the influence of CSR on organizational performance (Yoon and Chung, 2018; Ikram et al., 2019). In practice, more and more businesses realize that CSR can help them to be more sustainable and accountable (Ahmad et al., 2021). For example, in a Chinese firm, where current business scandals, labor issues, food safety, and environmental devaluation have brought about much negative reputation while simultaneously impacting performance negatively, there is a heightened need to establish business morals in the face of growing demand for CSR. Every business has to depend on society’s resources and deal with the communities for its growth. CSR is a concept that upholds and looks after the interests of both businesses and societies. CSR programs address the three major corporate wings: customers, society, and employees (Manzoor et al., 2019b).

Despite enormous efforts in studying CSR, a few critical issues require further attention. First, research on the relationship between CSR and firm reputation has produced inconclusive and contradictory findings. Some researchers have discovered a link between CSR and firm performance (Hou, 2019; Rehman et al., 2020; Bahta et al., 2021). Second, while much research has been done on the influence of CSR on organizational performance, little has been conducted to investigate the relationship between CSR and firm reputation and the intermediate process that links CSR and firm reputation. CSR benefits have also been positively associated with evidence of the entity’s success. As an example, consider the financial performance (Cho et al., 2019; Karyawati et al., 2020), increased customer confidence (Aljarah et al., 2018), massive and positive direct customer purchasing behavior (Zhang and Ahmad, 2021), raised in the level of complete stakeholder confidence (Del Brio and Bolaños, 2020), increase in employee job satisfaction (Farmaki et al., 2022). The domino effect of CSR on both performance and society suggests that CSR practices can occur and become powerful entities and indicators in determining the direction of long-term business success (Singh and Misra, 2021).

The necessary individual consequences highlighted in this research stream are CSR and organizational citizenship behavior (OCB), and firm reputation (Jacobsen and Beehr, 2022). Employee OCB can be understood as the voluntary participation of employees in different tasks of the organization, which do not fall under the ambit of their contracts. Few studies justify how and why CSR influences employees’ OCB (Oo et al., 2018; Supanti and Butcher, 2019). Furthermore, prior research has focused on the antecedents of OCB, but the association between OCB and a firm reputation in the business sector is still unknown. There are two opposing viewpoints on OCB, first is the optimistic view that says that OCB improves performance from a relational perspective (He et al., 2019; Yaakobi and Weisberg, 2020). Second, the opposing viewpoint contends that OCB places employees under stress and causes conflicts, resulting in poor performance (He et al., 2019; Fu et al., 2022), negative employee performance leads to a low level of firm reputation. As a result, it is worthwhile to investigate the relationship between OCB and the firm reputation further. CSR influences OCB and firm reputation by highlighting implied belongingness and the psychological link between the organization and the employees, as well as by fostering organizational identification in which “the individual defines himself or herself in terms of membership in a particular organization” (He et al., 2019).

This analysis aims to contribute to CSR research in the business sector. Further, prior research has not explored how certain CSR practices influence the development and implementation of organic organizational cultures. Furthermore, previous research has not provided empirical explanations for the relationships between CSR practices, firm reputation, and employee OCB. It is worth noting that one of the intangible assets in firm performance is the reputation (Pires and Trez, 2018), though reputation is the primary outcome of perceptions of a business’s CSR practices (González-Rodríguez et al., 2019). Even though firm reputation is an undeniable source of competitive advantage, only a few studies have looked into how CSR can be used as a tool to improve a firm reputation (Verčič and Ćorić, 2018; González-Rodríguez et al., 2019). Organizational culture is the characteristic and tangible personality of each organization. The organizational culture can effectively promote knowledge exchange, experience, and ideas (Asatiani et al., 2021). Meanwhile, organizational culture can provide a positive and better atmosphere/environment to facilitate the following organizational and individual outcomes. Organizations cannot survive or thrive in ever-changing environments unless their members act as good citizens by engaging in various positive behaviors (Isensee et al., 2020). OCB is an extra role behavior that is not specified or required by the formal job responsibilities (Celiker and Guzeller, 2022) and can be seen in an employee who voluntarily assists other employees in their work to promote the employer’s excellence without expecting to be compensated for it (Habeeb, 2019).

This study contributes to the literature on the line of CSR, organic organizational cultures, firm reputation, and OCB. A previous study by Agarwala et al. (2022) shows that CSR performance is still scarce in developing countries. This study would promote value to the current set of limited literature. Previous literature showed the direct influence of CSR on the firm reputation (Tangngisalu et al., 2020; Nguyen et al., 2021), as well as CSR’s impact on employee’s organizational citizenship behavior (He et al., 2019; Khaskheli et al., 2020) but this study expands these relationships *via* mediating mechanism of organic organizational cultures, which is an attractive contribution to the existing literature. Moreover, previous studies highlighted the direct effect of organic organizational cultures on firm performance (Noone et al., 2022), nevertheless, little research has studied the impact of organic organizational cultures on firm reputation and employee OCB. This study also covers these gaps by investigating the direct effect of organic organizational cultures on firm performance and employee OCB.

The main objective of this study was to unlock the effects of CSR on firm reputation and employee OCB in Pakistan. Therefore, we used organic organizational cultures (clan and adhocracy) as a mediator in this study. This study’s findings are the first of their kind in Pakistan. The study’s specific research questions are as follows:

1. Does CSR affect the firm reputation and employees’ OCB in medium and large enterprises in Pakistan?
2. Do organic organizational cultures (clan and adhocracy) mediate between CSR-firm reputation link and CSR-employee OCB linkage?
3. Can an employee’s OCB affect a firm reputation?

Following that, we provide a brief theoretical background and review of the literature, present the study’s methods and measures, and elaborate on the survey findings in detail. Our study concludes by discussing the research’s findings, limitations, and implications in the final section.

## 2. Theories and hypotheses development

The literature review relies on reputable scientific databases, including Wiley, Springer, Elsevier, Emerald, and Taylor and Francis. It covers the years 2017 to the present to establish current knowledge. Significant older research was included where it was necessary to present a comprehensive picture of the topic. We begin by establishing the overall context of the study. This research is based on three theories: the stakeholder theory, the social identity theory, and the social exchange theory.

The results of this study support the connections between CSR and the stakeholder theory by recognizing many stakeholder groups, including employees and the community. Advocates of CSR in modern business contend that organizations have obligations to stakeholders and their respective shareholders or investors. Employee attitudes and behaviors drive organizational culture and environment. CSR initiatives can encourage a culture of innovation (Chen, 2022). This study highlights the role of CSR in the organization’s culture and supports the stakeholder theory.

CSR can promote employee organizational identification (Martínez et al., 2014; Shin et al., 2016). Motivation and a desire to help others can make employees feel closer to their employer. Social identity theory provides evidence that CSR enhances organizational identification leading to OCB. In conformity with the social exchange theory, the leader and follower develop a reciprocal relationship in which one side receives something of value from the other and is compelled to respond in kind. Social exchange theory explains how CSR practices and firm reputation impact one another (Farooq et al., 2019). This study supports the social exchange hypothesis by highlighting the impact of CSR on corporate reputation and OCB.

As mentioned above, the theories employed for the purpose of this study are the stakeholder theory, the social identity theory, and the social exchange theory. The stakeholder theory argues that it is not only the shareholders that are impacted by firm decisions, but rather a several stakeholders including, but not limited to the employees, the customers, the suppliers as well as the communities. Based on this, our research involved employees in the process of data collection, since they are the most impacted by firm decisions. However, one limitation that we faced in employing this theory was that our data collection did not include the rest of the stakeholders.

The social identity theory explains individuals’ self-concept derived from perceived membership in a social group. Whereas the social exchange theory studies the interactions between individuals from a cost–benefit perspective. Our model is further supported by these two theories, to help explain the impact of CSR on OCB and FR. However, since we are focusing more on the impacts of CSR on OCB and FR, we could not dig deeper into individual employee self-identities.

### 2.1. Corporate social responsibility and firm reputation

Research demonstrates a positive link between CSR practices and FR (Jeffrey et al., 2018; Yadav et al., 2018; Park, 2019). Social exchange theory facilitates the connection between CSR perceptions and the trust (Tangngisalu et al., 2020). Each stakeholder’s overall impression of the company and level of trust can be characterized by the firm’s reputation (Yadav et al., 2018). Stakeholders are more likely to trust companies with strong CSR since high-quality management is an indicator. According to research, customers’ perceptions of a company’s CSR actions are positively correlated with their evaluations of its reputation (Arli et al., 2019). From the employee’s point of view, the employees’ impression of their organizations’ CSR is linked to their level of organizational commitment, which improves their firms’ reputation ratings (Lee and Tao, 2020). Among the company’s efforts to strengthen its reputation among senior-level executives, philanthropic activities may play a role (Özcan and Elçi, 2020). Under the theory of social exchange, the norm might regulate employee responses. A good perception of CSR is likely to boost employee confidence in their superiors, as employees see that the company has served the interests of all parties and so deserves greater trust. Businesses can enhance their firm reputation by focusing on proper CSR programs and communication channels (Ajayi and Mmutle, 2021). Stakeholders evaluate a firm’s reputation based on the signals they receive from the organization (Tangngisalu et al., 2020). In addition to the company’s financial performance and ownership, alerts based on philanthropic values used by the CSR contribute positively to CSR perception (Harun et al., 2020). CSR improves a business’s brand image and reputation, as well as its sales and customer loyalty, as well as its capacity to attract and retain employees (Bahta et al., 2021). Hence, based on arguments in the extant research, we propose the following hypothesis:

*H1*: CSR has a positive and significant effect on a firm reputation.

### 2.2. Corporate social responsibility and employee OCB

Voluntary individual behavior, not immediately or explicitly recognized by the formal reward system, and overall supports the efficient operation of the organization is referred to as organizational citizenship behavior (OCB; Bies, 1989). We have used social identity theory and social exchange theory to support the link between CSR-OCB. According to social identity theory, individuals classify themselves and share the benefits of this affiliation (Ashforth and Mael, 1989). Existing research has identified the favorable effects of organizational factors such as authentic leadership, organizational justice, and perceived organizational support on OCB among employees (Iqbal et al., 2018; Farid et al., 2019; Tran and Choi, 2019). Scientists have discovered a correlation between OCB and employee loyalty and identification with a firm (El-Kassar et al., 2021). CSR contributes to organizational identification and encourages employees to demonstrate extra-role and responsibility-taking behaviors (Wang et al., 2017). In this regard, employees who identify with their organization are more likely to maintain their self-identity through OCB (Wang et al., 2019; El-Kassar et al., 2021). Social exchange theory says that people trade with each other for both social and economic reasons (Blau, 1964). Characterized by obligations, trust, interpersonal attachments, or commitment to specific exchange partners (Lee, 2021), there is evidence that people are more likely to show OCB at work if they know that the company is socially responsible (Farid et al., 2019). Overall, the real-world data shows that CSR positively affects OCB. The social exchange theory explains why employees do things on their own time. When employees think a company’s CSR activities are fair, they behave cooperatively at work (Aftab et al., 2021). Also, when organizations help their employees socially and emotionally, the workers show their appreciation by trying to compensate for the good behavior they have received (Cappelli et al., 2020; Naz et al., 2020). In compliance with social exchange theory and social identity theory, and the information mentioned above from extant research, this study hypothesizes the following:

*H2*: CSR has a positive and significant effect on employees’ OCB.

### 2.3. Corporate social responsibility and organic organizational culture

In organizations in emerging nations, organic organizational cultures, such as clan and adhocracy, are standard (Awino, 2020). Due to their emphasis on flexibility, organic cultures are effective in these countries’ continuously changing and unpredictable environments (Alvesson and Lindkvist, 1993; Pasricha et al., 2018; Awino, 2020). Organizational culture is “a collective entity that emerges from individuals’ values and societal, ethical leadership, organic organizational cultures, and CSR (Schein, 2004; Žukauskas, 2018). Shared values “provide the normative or moral purpose of directing members in how to respond to certain crucial situations” (Ellemers et al., 2019). Li and Fung (2020) stated that values are ingrained in the layered creation of culture and should be analyzed to get insight into an organization’s culture.

By its stated objectives, the present study investigates organic organizational culture through the shared values of individuals in the organization. However, the importance of organic organizational culture in facilitating the understanding of diverse management processes has been widely demonstrated, for example, organizational innovation (Chen et al., 2018), competitive advantage (Ziaei Nafchi and Mohelská, 2020), and organizational effectiveness (Hassan, 2020; Khan et al., 2020). There is little interest in organic organizational culture in the search for CSR. The current study contributes to this research by investigating the critical role of organic organizational culture as a mediator in the relationship between CSR, firm reputation, and OCB. The findings of this study reinforce the links between CSR and stakeholder theory by identifying different stakeholder groups, namely employees and society (Dmytriyev et al., 2021). Contemporary advocates of the CSR (Waheed and Zhang, 2020) argue that business organizations have a responsibility not only to investors or shareholders but also to stakeholders, i.e., employees and society.

#### 2.3.1. CSR and the clan culture

According to Alharbi and Abedelrahim (2018), distinctive features of clan-type companies are teamwork, employee engagement programs, and the company’s commitment to the employee. Some of the basic assumptions in clan culture are that the environment can be better managed through teamwork and employee development, that customers are better treated as partners, and that the organization creates a humane work environment (Acquah et al., 2020; Nanayakkara and Wilkinson, 2021; Zhang et al., 2022). Firms are responsible to their respective investors, shareholders, and stakeholders (Waheed and Zhang, 2020). CSR improves the corporate image (Kim et al., 2020). A positive firm reputation is associated with increased job satisfaction and decreased employee turnover (Chatzopoulou et al., 2021). The organization of clan culture is held together by loyalty and tradition (Kheir-Faddul et al., 2019). CSR can increase employee loyalty to the organization (Stojanovic et al., 2020). Hence, we posit that:

*H3*: CSR positively and significantly affects clan culture (organic organizational culture).

#### 2.3.2. CSR and the adhocracy culture

Adhocracy culture is a developmental, organizational culture that emphasizes the development, growth, innovation, and productivity of new products and services (Misigo et al., 2019). An adhocracy culture is characterized by a dynamic, entrepreneurial, and creative environment (Scaliza et al., 2022). Strategic plans for a company with an adhocracy culture are based on the desire for constant change and acquiring new knowledge and resources. Organizations that deal with an online business, defined as a new economy using modern technology, are examples of this culture (Acar and Acar, 2014; Misigo et al., 2019; Zeb et al., 2021). Innovativeness and ever-improving eminence of a company’s products and services are granted prominence in adhocracy culture. CSR programs can assist in fostering a culture of innovation (Chen, 2022). Hence, we propose the following hypothesis based on arguments in the extant research.

*H4*: CSR positively and significantly affects Adhocracy culture (organizational culture).

### 2.4. Organizational culture on firm reputation

Organic organizations are adaptable and flexible (Hartnell et al., 2019) allowing them to be more responsive to the market (Pasricha et al., 2018). Organic cultures promote risk-taking, nurture employees’ aspirations, and provide a collaborative environment, which can be the basis for competitive advantage and help a business grow and improve its chances of success (Dimitrova, 2018). A good firm’s reputation is created following a firm’s success and destroyed following a firm’s failure (Tadelis, 2003). The employees of an organization influence how projects are perceived and implemented, as well as how clients, partners, and the general public perceive the organization (Afsar et al., 2020). Consequently, the need to enhance a firm reputation has prompted businesses to examine their culture and adopt ways to evolve and improve it (Hatch and Schultz, 1997).

Clan culture is frequently characterized as a particularly welcoming workplace (Xie et al., 2020; Li et al., 2021; Shaked, 2021; Huang et al., 2022). It resembles a large family. The leaders, or the heads of the organization, are seen as mentors and possibly even parents. The organization is held together by tradition or loyalty, and high commitment exists. The organization prioritizes the long-term value of human resource development and places a premium on teamwork and morale. Success is defined by customer awareness and concern for customers. Employee trust is typically related to clan culture (Masood et al., 2006). Ideally, increasing employee trust increases the company’s reputation among its workforce (Yadav et al., 2018). It has been hypothesized that employee trust leads to good attitudes, such as dedication and job satisfaction, and behavior, such as increased effort (Michaelis et al., 2009). Company culture and identity, contact personnel, and physical environment are significant factors affecting consumers’ perceptions of a firm reputation. Workplaces characterized by an adhocracy culture are entrepreneurial and inventive (Misigo et al., 2019; Noone et al., 2022). Adhocracy culture is viewed as a culture that fosters innovation (Hamzah et al., 2022). Moreover, Aladwan and Alshami (2021) found that invention significantly impacts a company’s reputation. Hence the following hypothesis is proposed:

*H5*: Clan culture has a positive effect on a firm reputation.

*H6*: Adhocracy culture has a positive and significant effect on a firm reputation

### 2.5. Organizational culture on OCB

Clan culture stresses collaboration among members, including a family-like atmosphere, morale, communication, and cohesion, and focuses on maintaining and enhancing human relationships inside the organization (Jeong et al., 2019). Adhocracy culture promotes organizational success through creativity, innovation, and challenges, which also leads to the acquisition of new resources and entrepreneurship (Azeem et al., 2021). Organizational culture influences employee behavior significantly (Kawiana et al., 2018), so there is likely a favorable relationship between these organic organizational cultures and OCB. Indeed some studies (Zeyada, 2018; Mitonga-Monga, 2019; Mitonga-Monga and Flotman, 2021) found an increase in OCB among employees who were linked with and understood the organizational principles and ethics. In addition, they concluded that support, structure, and risk tolerance were among the most influential contributors to organizational culture on employee OCB. OCB can be influenced by the degree of commitment, mutual trust, and shared values among the organization’s employees (Bhoki, 2020). Additionally, an innovative work atmosphere can affect OCB (Soelton et al., 2020). Taking into account these relationships, some researchers assessed the impact of different organizational cultures on the OCB (Al-Shurafat and Halim, 2018; Mansouri et al., 2018; Lockhart et al., 2020). Hence our study offered the following hypotheses:

*H7*: Clan culture has a positive and significant effect on OCB.

*H8*: Adhocracy culture has a positive and significant effect on OCB.

### 2.6. OCB and firm reputation

Firm reputation is an intangible resource that gives the company a competitive advantage because it affects customer loyalty and financial outcomes (Farhan et al., 2020; Islam et al., 2021; Khan et al., 2022; Le, 2022). A good firm’s reputation could reduce the cost for sellers and buyers to do business with each other since most people prefer to do business with people who have already shown they can be trusted (Wiedmann et al., 2013). Existing research has looked at a firm’s reputation from the perspective of different stakeholders, such as customers (Osakwe et al., 2020) and potential employees (Tangngisalu et al., 2020). Little attention has been paid to employees, a potentially vital part of a firm’s reputation program, and critical employee outcomes, particularly their OCB. González-Rodríguez et al. (2019) says that a firm’s reputation is the socially shared impression of how people see the firm. Similarly, employees of the same company share their images of their employer. Based on what the public thinks, employees know how trustworthy the company they work for is as proposed by social identity theory (SIT), which pertains to a person’s oneness with a group (an individual’s sense of belonging to a group) and is a significant factor in their behavior (Ashforth and Mael, 1989). Hence, based on arguments in the extant research, we propose the following hypothesis:

*H9*: Employee’s OCB positively and significantly affects a firm’s reputation.

### 2.7. Mediating mechanism of organic organizational culture (CC&AC)

Though, a few studies have established associations between organizational culture and CSR other than the Pakistani medium and large enterprises without the inclusion of any moderation or mediation variable (El-Kassar et al., 2021). CSR is a business paradigm in which businesses make a concentrated effort to operate in ways that benefit society and the business environment rather than harm them (Kapelus, 2002), as well as CSR, is a source of employee satisfaction (Wisse et al., 2018). Regarding CSR, it determined that integration of CSR competence may help to achieve company performance and satisfied employees (Wang et al., 2020). Furthermore, many academicians and specialists have urged focusing more on CSR, especially in light of empirical studies conducted in developing countries (e.g., Manzoor et al., 2019b; Wang et al., 2020). Researchers should concentrate on developing countries because CSR research in developed countries has been extensive in recent decades (El-Bassiouny and El-Bassiouny, 2018; Kudłak et al., 2018; Nill and Papp, 2020).

**Figure 1:**
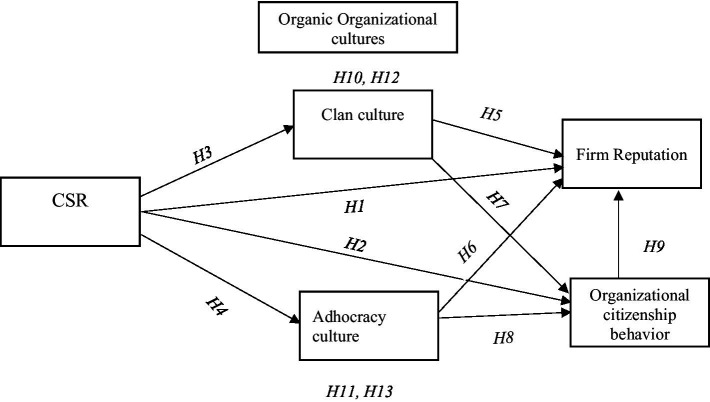
Conceptual framework of the study.

Nevertheless, no well-known work has determined the nexuses between CSR, firm reputation, and OCB by employing organic organizational culture (clan and adhocracy culture) as the mediator in underdeveloped countries’ medium and large enterprises. As a result, based on the arguments mentioned above and hypothesized relationships, we seek to specify empirical and analyzed proof discovering the mediating mechanism of organic organizational culture (clan and adhocracy culture) in the relationships between CSR firm reputation and OCB (see Figure 1). Accordingly, we suppose that:

*H10*: Clan culture has a positive mediating effect on the nexus of CSR and firm reputation.

*H11*: Adhocracy culture has a positive mediating effect on the nexus of CSR and firm reputation.

*H12*: Clan culture has a positive mediating effect on the nexus of CSR and OCB.

*H13*: Adhocracy culture has a positive mediating effect on the nexus of CSR and OCB.

## 3. Methods of the study

The cross-sectional design and self-administered questionnaires were used to collect primary data from employees working in various medium and large enterprises in Pakistan. The reason for using questionnaires as the main data source was that our research has been conducted in an undocumented market of Pakistan, where secondary data is often not available. In such markets, primary research is often the most reliable source. The questionnaire adopted constructs on CSR from Manzoor et al. (2019b)‘s study, those on OOC from Cameron and Quinn (2011)‘s study, while the constructs on Firm Reputation and OCB were taken from Tangngisalu et al. (2020) and Yang et al. (2022)‘s studies, respectively.

The enterprises from whose employee data was collected are big shopping malls, restaurants, Hotels, Automobiles, and pharmaceutical companies in urban areas of Pakistan. We visited these enterprises to highlight the significance of the study and encourage workers to contribute; questionnaires were circulated among them. These questionnaires consisted of the entire demographic characteristics of the participants and all main study variables (CSR, organic organizational cultures (Clan and Adhocracy), organizational citizenship behavior, firm reputation, etc.).

For this study, convenience sampling was used. We used this sampling technique to recruit participants who are convenient and easily accessible. We distributed 500 questionnaires to owners, top-level management workers, and employees of these enterprises, and 360 (72%) filled questionnaires were returned for analysis. The demographic characteristics of respondents are shown in Table 1.

**Table 1:** Demographic statistics of study participants (*n* = 360).

Demographics descriptions	No/Percentage (%)
**Gender**	
Male	257 (71.4)
Female	103 (28.6)
**Age**	
30 and below	113 (31.4)
31–40	148 (41.1)
41–50	63 (17.5)
51–60	27 (7.5)
Above 60	09 (2.5)
**Large and medium enterprises**	
Shopping Malls	91 (25.3)
Pharmaceutical Firms	104 (28.9)
Hotels/Restaurants	95 (26.4)
Automobiles/Textile Enterprises	70 (19.4)
**Qualification**	
Professional Degree	86 (23.9)
Master’s degree	142 (39.4)
Bachelor’s degree	91 (25.3)
Other	41 (11.4)

### 3.1. Measurement instruments of the study variables

Standard scales from the existing literature were used for the study measures. These measures’ items were anchored on a 5-point Likert scale ranging from 1 to 5, with 1 being strongly disagreed and 5 strongly agreeing.

- Corporate Social Responsibility: The questionnaire on the CSR variable consisted of 16 items and the CSR questionnaire is adopted from the previous study by Manzoor et al. (2019b).
- Organic organizational cultures: We focused on two Organic organizational cultures, i.e., (a) Clan and (b) Adhocracy. Each of the constructs has six measuring items founded on Cameron and Quinn (2011) Organizational Culture Assessment Instrument (OCAI). These culture types are determined by the characteristics of the organization, organizational leadership, employee management, organizational glue, strategic emphasis, and success criteria (Cameron and Quinn, 2011).
- Firm reputation: Three questions refer to the firm reputation variable, which we adopted from a previous study by Tangngisalu et al. (2020). The same questions are used in earlier literature (Rupp et al., 2006; Lai et al., 2010).
- Organizational citizenship behavior: In this study, we employed the OCB scale established by Yang et al. (2022), and previously used in the literature (Bachrach et al., 2007). Total of 10 items using the Likert five-point scale measurement, the score was from 1 to 5, indicating “strongly disagree” to “strongly agree.”

## 4. Empirical results and findings

We used SPSS (v.26) and smart PLS (v.3.3.3) for analyses. The structural equation modeling technique (SEM) was applied for the mediation approach. PLS-SEM includes the measurement model description to validate the basic structure of the variables in the suggested model. Path analysis is used to scrutinize the study hypotheses demonstrated in the model. The variables with good factor loading convergent validity, composite reliability (CR), and discriminant validity were conducted for further analysis using the measurement model specification.

Furthermore, discriminant validity was used to calculate the associations between latent constructs by comparing the differences between latent variables that were validated using experts’ recommended criteria (Manzoor et al., 2021). Structural model valuation weighs path coefficients and tests their significance. Model fit for SEM was exposed using the goodness technique of Hu and Bentler (1999) by Standardized Root Mean Square Residual (SRMR). It is suggested that SRMR values should not surpass 0.08 (Hu and Bentler, 1999). All of the techniques applied are well-matched with previous research studies in the area; therefore, scholars are now encouraged to generate outcomes by employing these tools and techniques (Pasricha et al., 2018; Dana et al., 2022).

### 4.1. Descriptive measurements

Table 2 displays the study constructs’ means, standard deviations, and correlations. The correlation coefficients revealed that all study constructs are highly correlated.

**Table 2:** Descriptive measurements and Pearson correlation of the constructs.

Constructs (*n* = 360)	Mean	Std. D	Correlations of study constructs				
			1	2	3	4	5
1. CSR	3.942	0.754	1				
2. Clan Cultural	3.938	0.779	0.471**	1			
3. Adhocracy Culture	3.865	0.909	0.520**	0.458**	1		
4. Firm reputation	3.901	0.897	0.424**	0.441**	0.463**	1	
5. OCB	3.951	0.945	0.523**	0.425**	0.399**	0.397**	1

^**^Correlation is significant at the 0.01 level (two-tailed).

### 4.2. Common method bias

This study’s measurement items were examined for common method bias (CMB; Manzoor et al., 2019a). Harman’s single factor analysis was used to check for measurement biases (Podsakoff and Organ, 1986), which exposed no CMB issue in the study’s data as the total variance obtained by one factor is 42.3% and it is lower than the suggested threshold of 50%.

### 4.3. Measurement model estimation

The measurement model evaluation is based on the recommendations of Hair et al. (2006) to verify the reliability and validity of the variables. All included 41 measures were estimated whole from elimination as the scrutinized factor loadings made over the recommended value of 0.60. The factor loadings, alpha coefficient, Composite Reliability (CR), and Average Variance Extracted (AVE) are all recorded in Table 3. The AVE and CR of all the indicators are higher than 0.50 and 0.70, correspondingly as per the recommended cut-off by specialists (Hair et al., 1998; Rabiul et al., 2021). As a result, reliability and convergent validity are confirmed. Likewise, discriminant validity is recognized, as listed in Table 4, using the criterion proposed by Fornell and Larcker (1981). Oliveira et al. (2016) advised that discriminant validity may be acquired by comparing interrelations of the variables with √AVEs. It is recommended that the values of √AVE be greater than the values of the following interconnections of the constructs. Consequently, values on the following model show the presence of such validity.

**Table 3:** Factor loading and reliability.

Construct and items	Factor loading	Cronbach’s alpha/CR	AVE
**Corporate social responsibility**		0.965/0.968	0.658
CSR1	0.818		
CSR2	O.814		
CSR3	0.767		
CSR4	0.752		
CSR5	0.815		
CSR6	0.814		
CSR7	0.856		
CSR8	0.846		
CSR9	0.739		
CSR10	0.813		
CSR11	0.830		
CSR12	0.851		
CSR13	0.824		
CSR14	0.793		
CSR15	0.828		
CSR16	0.807		
**Clan culture**		0.898/0.921	0.662
CC1	0.829		
CC2	0.779		
CC3	0.856		
CC4	0.769		
CC5	0.859		
CC6	0.784		
**Adhocracy culture**		0.926/0.942	0.730
AC1	0.890		
AC2	0.856		
AC3	0.862		
AC4	0.867		
AC5	0.810		
AC6	0.841		
**Firm reputation**		0.752/0.858	0.668
FR1	0.830		
FR2	0.831		
FR3	0.791		
**Organizational citizenship behavior**		0.957/0.963	0.722
OCB1	0.852		
OCB2	0.899		
OCB3	0.892		
OCB4	0.836		
OCB5	0.795		
OCB6	0.843		
OCB7	0.894		
OCB8	0.892		
OCB9	0.837		
OCB10	0.745		

**Table 4:** Discriminant validity (Fornell and Larcker criterion).

	CSR	CC	AC	FR	OCB
CSR	**0.814**				
CC	0.474	**0.854**			
AC	0.522	0.465	**0.811**		
FR	0.428	0.488	0.464	**0.817**	
OCB	0.530	0.435	0.435	0.404	**0.850**

Discriminant validity is shown in bold on the diagonal, whereas the other values indicate correlations with other constructs. CSR, corporate social responsibility; CC, clan culture; AC, adhocracy culture; FR, firm reputation; OCB, organizational citizenship behavior.

### 4.4. Hypothesized path evaluation

Tables 5, 6 describe the outcomes of the SEM evaluation, which was used to test the hypotheses of the study. The assessment of direct effects is shown in Table 5. The current SRMR value is 0.056, which is in fulfillment with the emphasized condition. The SEM outcomes validated CSR’s significant and positive impact on firm reputation as established by *β* = 0.14 at *p* < 0.05. Therefore, hypothesis 1 was endorsed. Then, hypothesis 2 is predicted with the relationship between CSR and OCB. We found support for hypothesis 2 (*β* = 0.46, *p* < 0.001). Furthermore, hypothesis 3 postulates the relationship between CSR and clan culture. Which was endorsed as CSR exposed to have a positive and significant influence on clan culture (*β* = 0.48, *p* < 0.001). It is assumed in hypothesis 4 that adhocracy culture positively affects CSR. We found complete approval for hypothesis 4. In accordance with standardized (*β* = 0.62, *p* < 0.001), significant and strong relationship between adhocracy culture and CSR was recognized. According to hypothesis 5, clan culture and firm reputation have a positive relation. As revealed in Table 5, clan culture has a significant and positive relationship with firm reputation (*β* = 0.24, *p* < 0.001), confirming hypothesis 5. Likewise, hypothesis 6 assumed there is a significant and optimistic relationship between clan culture and OCB. This study found support for hypothesis 6 (*β* = 24, *p* < 0.001). Then, hypothesis 7 is expected with the connection between the adhocracy culture and firm reputation. We found support for hypothesis 7 (*β* = 0.24, *p* < 0.001). likewise, hypothesis 8 postulated with the relationship between OCB and adhocracy culture was confirmed as adhocracy culture was exposed to have a positive and significant influence on OCB (*β* = 0.12, *p* < 0.05). Similarly, hypothesis 9 assumed a positive and significant association between OCB and firm reputation. This study found support for hypothesis 9 (*β* = 0.13, *p* < 0.001), as shown in Table 5.

**Table 5:** Results of the path coefficient of the structural model (direct relationships).

Hypotheses	Relationship	(*β*) estimates	St. Error	*p*-value	Decision
H1	CSR → FR	0.145	0.068	0.035	Supported
H2	CSR → OCB	0.463	0.067	0.000	Supported
H3	CSR → CC	0.486	0.048	0.000	Supported
H4	CSR → AC	0.627	0.054	0.000	Supported
H5	CC → FR	0.241	0.061	0.000	Supported
H6	CC → OCB	0.240	0.062	0.000	Supported
H7	AC → FR	0.243	0.053	0.000	Supported
H8	AC → OCB	0.121	0.055	0.030	Supported
H9	OCB → FR	0.138	0.051	0.007	Supported
*CC*	*R*^2^ = 0.22				
*AC*	*R*^2^ = 0.27				
*FR*	*R*^2^ = 0.32				
*OCB*	*R*^2^ = 033				
SRMR	0.056				

**Table 6:** Results of bootstrapping for the direct, indirect, and total effect of the hypothesized model.

Total effects				Direct effects			Indirect effects			
	*β*	*T*-value	*p*	*β*	*T*-value	*p*	Hypotheses of Mediation	*β*	*T*-value	*p*-value
CSR → FR	0.42	6.27	0.000	0.12	1.27	0.203	H10: CSR → CC → FR	0.101	2.275	0.023
CSR → OCB	0.53	9.70	0.000	0.37	5.23	0.000	H11: CSR → AC → FR	0.126	2.151	0.036
CSR → CC	0.47	6.43	0.000	0.47	6.43	0.000	H12: CSR → CC → OCB	0.097	2.566	0.010
CSR → AC	0.52	7.89	0.000	0.52	7.89	0.000	H13: CSR → AC → OCB	0.062	1.762	0.078
CC → FR	0.24	2.79	0.005	0.21	2.41	0.016				
CC → OCB	0.20	2.77	0.006	0.20	2.77	0.006				
AC → FR	0.25	2.46	0.014	0.24	2.25	0.023				
AC → OCB	0.11	1.84	0.065	0.11	1.84	0.065				
OCB → FR	0.14	1.97	0.049	0.14	1.97	0.049				

CSR, corporate social responsibility; CC, clan culture; AC, adhocracy culture; FR, firm reputation; OCB, organizational citizenship behavior.

### 4.5. Mediation or indirect effects

An assessment of indirect effects is given in Table 6. It concerned the execution of the mediation analysis according to the method suggested by Baron and Kenny (1986). This technique recommends that the explanatory variable be related to the outcome variable, that the explanatory variable be linked with the mediator, that the mediator is connected to the predicted construct, and that the presence of the mediator should be lower (partial mediation) or render insignificant (complete mediation) the explanatory variable’s prior direct relationship with the outcome variable.

Hypotheses *H*10–*H*13 investigate the influence of CSR on firm reputation and OCB *via* the organic organizational culture (clan and adhocracy), respectively. Support for hypotheses *H*1–*H*9 proved the first three conditions, as suggested by Baron and Kenny. The next critical phase was to evaluate the effect of clan and adhocracy cultures on the association between CSR, firm reputation, and OCB.

To assess full or partial mediation, we executed percentile bootstrapping and bias-corrected bootstrapping at a 95% confidence interval with 5,000 bootstrap samples (Kozhakhmet and Nurgabdeshov, 2022). To test the significance of indirect effects as Preacher and Hayes (2008), we estimated the confidence of the interval of the lower and upper bounds. As shown in Table 6, we discovered that the indirect effects of clan culture on firm reputation (*β* = 0.10, *p* < 0.05, *T* = 2.27) and OCB (*β* = 0.09, *p* < 0.05, *T* = 2.56) are significant. Similarly, the indirect effects of adhocracy culture on firm reputation (*β* = 0.12, *p* < 0.05, *T* = 2.15) and OCB (*β* = 0.06, *p* < 0.1, *T* = 1.76) are significant. The direct connection between CSR and firm reputation (*β* = 0.12, *p* = 0.20, and *T* = 1.27) is insignificant and supports hypotheses 10 and 11 with complete mediation. Furthermore, the direct connection between CSR and OCB (*β* = 0.37, *p* < 0.001, and *T* = 5.23) is significant and endorsed hypotheses 12 and 13 with partial mediation. The findings reveal the partial mediation role of clan and adhocracy cultures in the CSR - OCB connection.

**Figure 2:**
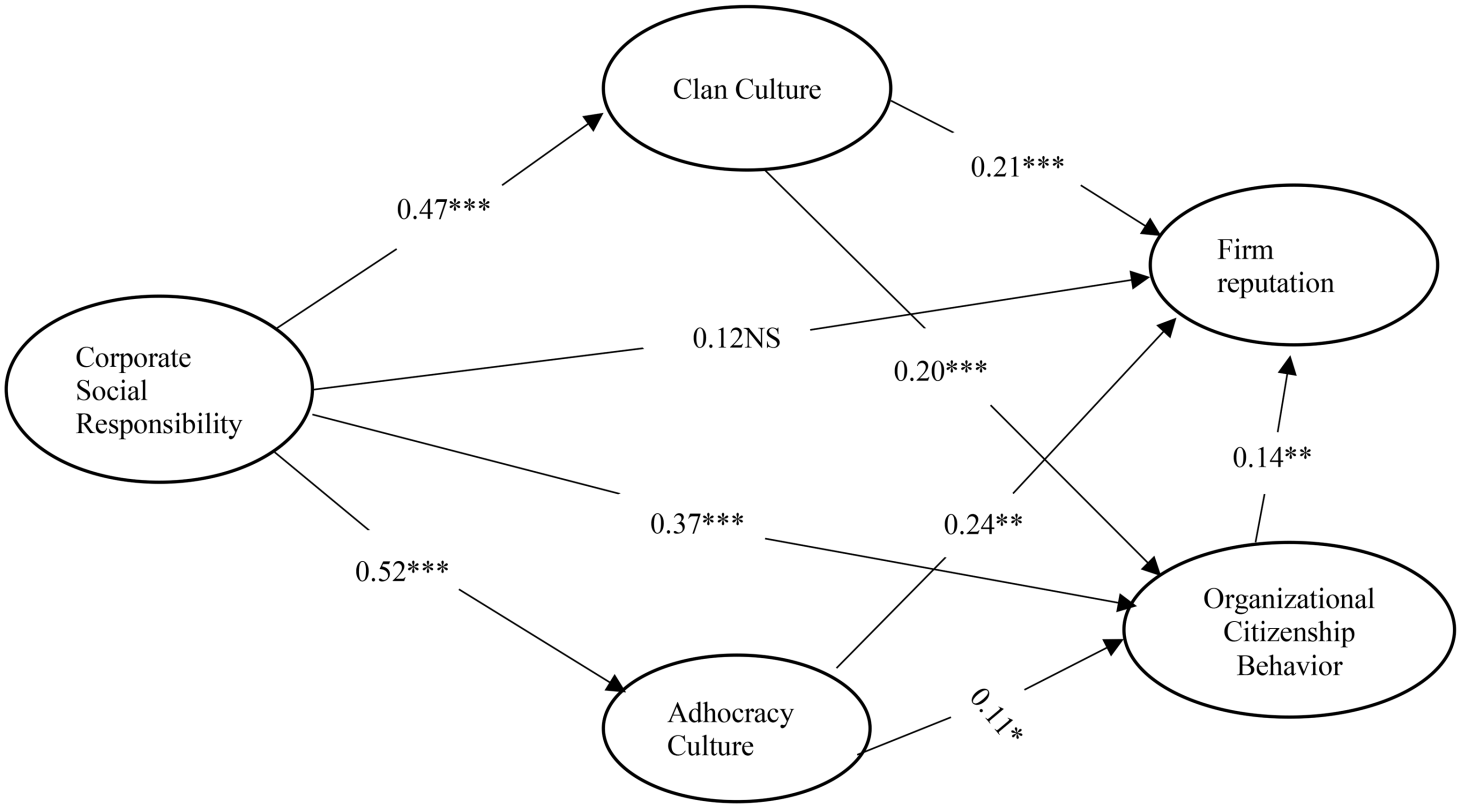
SEM modeling. significant at ***p < 0.01 and **p < 0.05.

An additional endeavor was undertaken, involving an examination of the synchronized influence of clan and adhocracy cultures on the (i) CSR–firm reputation linkage and (ii) CSR–OCB linkage was made. Figure 2 illustrates the structural model that demonstrates this.

Figure 2 demonstrates the SEM results, which show that the path from CSR to clan culture is significant (*β* = 0.47; *p* < 0.01) and that the paths from clan culture to firm reputation (*β* = 0.21; *p* < 0.01) and to OCB (*β* = 0.20; *p* < 0.01) are also positive and show significant relations. Figure 2 shows that the direct path from CSR to firm reputation (*β* = 0.12, *p* > 0.05) is insignificant, confirming full mediation, whereas the direct path from CSR to OCB (*β* = 0.37, *p* < 0.01) is significant demonstrating partial mediation. Likewise, the path from CSR to adhocracy culture is significant (*β* = 0.52; *p* < 0.01), as are the paths from adhocracy culture to firm reputation (*β* = 0.24; *p* < 0.01) and to OCB (*β* = 0.11; *p* < 0.05) are also significant and displays positive connections. According to Figure 2, the direct path from CSR to firm reputation is insignificant, confirming full mediation, whereas the direct path from CSR to OCB is significant, proving partial mediation.

## 5. Discussion

The prime aim of this study was to test the empirical relations between CSR and firm reputation and organizational citizenship behavior in the existence of organic organizational cultures as mediators between Pakistani large and medium enterprise employees. The study’s results also endorse previous research studies in which mediating mechanisms of organic organizational cultures (clan and adhocracy) between CSR and firm reputation were discovered (Balaji et al., 2020; Sackmann, 2021; Arabeche et al., 2022). It is proven that workers who have CSR activities in their enterprises have inspiring spirits towards an appealing goal and experience an excellent level of satisfaction and alternatively possess commitment, i.e., OCB. These findings show positive relationships between organic organizational cultures (clan and adhocracy), firm reputation, and OCB (Sackmann, 2021; Arabeche et al., 2022; Youn and Kim, 2022). Employees with a better understanding of clan and adhocracy culture are happier and more committed to their organization/enterprise.

The main finding of this study is that organic organizational cultures act as mediators in the relationship between CSR and firm reputation and OCB. Leaders and top management who want to improve the firm reputation and boost OCB among employees must be capable of setting up an ethical and organic organizational culture. The study’s findings supported all of the hypotheses depicted in the proposed model. This study demonstrates that CSR has a significant direct effect on firm reputation and OCB, as well as an indirect effect on firm reputation and OCB *via* organic organizational cultures. The indirect impact of CSR *via* clan and adhocracy cultures provides more insight into the relationship between CSR, firm reputation, and OCB. Both the clan and adhocracy cultures were discovered to fully mediate the CSR-firm reputation and partially mediate the relationship of CSR-OCB. This mediating mechanism of organic organizational culture significantly contributes toward linking CSR and the firm reputation (Ikram et al., 2019).

This study explains the association between CSR, organic organizational cultures, firm reputation, and OCB, particularly in large and medium enterprises, a previously unexplored sector. It provides preliminary support for the critical role of organic cultures in expanding top management competence as a driver of CSR in large and medium-sized enterprises. It proposes to help these enterprises’ managers by increasing their knowledge of culture types, which are critical to be confident to cope with the organization’s responsiveness to its stakeholders and thus accelerate organizational performance and reputation. Aside from that, the study strengthens our knowledge of the absence of mutual exclusivity between different types of organic organizational culture (Bhoki, 2020). It highlights the importance of cultural complementarity in achieving desired organizational outcomes by highlighting the coexistence of organic clan and adhocracy cultures as encouraging the CSR-firm reputation relation and the CSR-OCB link.

Furthermore, the study findings are consistent with previous research linking CSR and firm reputation (Ikram et al., 2019; Tangngisalu et al., 2020) as well as CSR and OCB (Oo et al., 2018; Youn and Kim, 2022). According to the current study, this dynamic influence is more marked in large and medium-sized businesses because managers in these organizations are mainly responsible for a diverse set of stakeholders and are apprehensive about achieving the organization’s social mission. As a result, prior research indicates that an organization’s leadership impacts organizational outcomes through CSR. The study contends that organizational culture plays a role in this impact because, according to the results, the behavior of the leader defines the organization’s culture, which describes the CSR strategies of the organization.

### 5.1. Implications, limitations, and future directions of research

This study has significant implications for medium and larger enterprises regarding the relevance of CSR in terms of the effectiveness of such organizations. The study findings reveal how CSR techniques and qualities support a firm reputation by demonstrating the significant mediator role of organic organizational cultures. Implementing CSR qualities increases employee job satisfaction, which improves the firm’s reputation and employee OCB. Another practical implication of this study is that top-level management, such as leaders and managers, should be trained in managing organic cultures, which will improve their performance, organizational performance, and firm reputation. In conclusion, our research suggests that the primary goal of training should be to increase employee job satisfaction and performance to achieve high organizational outcomes and benefits. They are also directed to incorporate social responsibility agendas, as this can help enhance individuals’ strengths and nurture CSR-friendly value systems in the organization. Additionally, this would increase the performance of the personnel and thus inclusive organizational performance. Furthermore, the study findings provide critical efforts to specialists in the state’s enterprise area by facilitating a better understanding of medium and large enterprise management. Because Pakistan is a developing country, the study believes that these results and their implications are also relevant to other developing economies.

It is critical to highlight the study’s few limitations, which may lead to future investigation. The cross-sectional research design is applied for data collection. A future study could present the research model using a longitudinal study technique to avoid the ambiguity of a causal correlation. Second, the current study is limited to medium and large businesses. As a result, it is suggested that the study be expanded to include other organizations to overcome the limitations of this study. Third, this study was conducted in Pakistan. Future research studies should be conducted in other less-developed countries to investigate the model and increase the generalizability of the results. Finally, the other possible moderating and mediating effects of certain variables on the CSR–firm performance linkage and CSR–OCB link can be further studied.

## 6. Conclusion

This empirical study elaborated on the relationship between CSR and firm reputation, organizational citizenship behavior, and the mediation mechanism of organic organizational cultures, i.e., (clan and adhocracy). This study found that organic organizational culture (clan and adhocracy) fully mediated CSR’s impact on a firm’s reputation and partially mediated the relationship with OCB. Top management and enterprise leaders can improve CSR activities and develop organic organizational cultures by empowering their employees. Taken together, these results deliver more comprehension of CSR and OCB research and propose various phases that can be taken to promote it in larger enterprises. Finally, it offers important insights for businesses seeking to develop socially responsible and improve their potential for enhancing firm reputation. Despite the limitations of our study, we hope it will serve as a solid foundation for upcoming studies.

## Data availability statement

The raw data supporting the conclusions of this article will be made available by the authors, without undue reservation.

## Ethics statement

The studies involving human participants were reviewed and approved by University of International Business and Economics, Beijing China. The patients/participants provided their written informed consent to participate in this study.

## Author contributions

HA initiated the basic idea and wrote the manuscript and built the article structure. JY reviewed and improved the manuscript. FM reviewed and improved the methodology of this study. AM further reviewed and improved methodology and improved structure. All authors contributed to the article and approved the submitted version.

## Conflict of interest

The authors declare that the research was conducted in the absence of any commercial or financial relationships that could be construed as a potential conflict of interest.

## Publisher’s note

All claims expressed in this article are solely those of the authors and do not necessarily represent those of their affiliated organizations, or those of the publisher, the editors and the reviewers. Any product that may be evaluated in this article, or claim that may be made by its manufacturer, is not guaranteed or endorsed by the publisher.

## References

[ref1] Acar A. Z. Acar P. (2014). Organizational culture types and their effects on organizational performance in Turkish hospitals. Emerg. Mark. J. 3, 18–31. doi: 10.5195/EMAJ.2014.47

[ref2] Acquah H. E. Sarkodie N. A. Enoch B. Adams L. Djanie B. N. A. Nunoo J. (2020). Influence of organisational culture on employee commitment: evidence from environmental protection agency in Ghana. Int. J. Technol. Manag. Res. 5, 45–57. doi: 10.47127/ijtmr.v5i3.100

[ref3] Afsar B. Al-Ghazali B. M. Rehman Z. U. Umrani W. A. (2020). Retracted: the moderating effects of employee corporate social responsibility motive attributions (substantive and symbolic) between corporate social responsibility perceptions and voluntary pro-environmental behavior. Corp. Soc. Responsib. Environ. Manag. 27, 769–785. doi: 10.1002/csr.1843

[ref4] Aftab J. Sarwar H. Amin A. Kiran A. (2021). Does CSR mediate the nexus of ethical leadership and employee’s job performance? Evidence from North Italy SMEs. Soc. Responsibility J. doi: 10.1108/SRJ-09-2020-0393

[ref5] Agarwala N. Pareek R. Sahu T. N. (2022). Does board independence influence CSR performance? A GMM-based dynamic panel data approach. Soc. Responsibility J. doi: 10.1108/SRJ-10-2020-0433

[ref6] Ahmad N. Ullah Z. Mahmood A. Ariza-Montes A. Vega-Muñoz A. Han H. (2021). Corporate social responsibility at the micro-level as a “new organizational value” for sustainability: are females more aligned towards it? Int. J. Environ. Res. Public Health 18:2165. doi: 10.3390/ijerph18042165, PMID: 33672139 PMC7927048

[ref7] Ajayi O. A. Mmutle T. (2021). Corporate reputation through strategic communication of corporate social responsibility. Corporate Commun. Int. J. 26, 1–15. doi: 10.1108/CCIJ-02-2020-0047

[ref8] Aladwan S. A. Alshami S. I. (2021). The impact of service excellence and service innovation on organisational reputation: quantitative evidence from Jordanian public sector. TQM J. 33, 1544–1560. doi: 10.1108/TQM-05-2020-0117

[ref9] Alharbi S. H. Abedelrahim S. (2018). Organizational culture assessment using the competing values framework (CVF) in public universities in Saudi Arabia: a case study of Tabuk university. Int. J. Bus. Manag. 6, 1–16. doi: 10.20472/BM.2018.6.2.001

[ref10] Aljarah A. Emeagwali L. Ibrahim B. Ababneh B. (2018). Does corporate social responsibility really increase customer relationship quality? A meta-analytic review. Soc. Responsibility J. 16, 28–49. doi: 10.1108/SRJ-08-2018-0205

[ref11] Al-Shurafat M. S. Halim B. B. A. (2018). A review of organisational culture and organizational commitment. IOSR J. Bus. Manag. 20, 21–26. doi: 10.9790/487X-2003052126

[ref12] Alvesson M. Lindkvist L. (1993). Transaction costs, clans and corporate culture. J. Manag. Stud. 30, 428–453.

[ref13] Arabeche Z. Soudani A. Brahmi M. Aldieri L. Vinci C. P. Abdelli M. E. A. (2022). Entrepreneurial orientation, organizational culture and business performance in SMEs: evidence from emerging economy. Sustainability 14:5160. doi: 10.3390/su14095160

[ref14] Arli D. Van Esch P. Northey G. Lee M. S. Dimitriu R. (2019). Hypocrisy, skepticism, and reputation: the mediating role of corporate social responsibility. Mark. Intell. Plan. doi: 10.1108/MIP-10-2018-0434

[ref15] Asatiani A. Hämäläinen J. Penttinen E. Rossi M. (2021). Constructing continuity across the organisational culture boundary in a highly virtual work environment. Inf. Syst. J. 31, 62–93. doi: 10.1111/isj.12293

[ref16] Ashforth B. E. Mael F. (1989). Social identity theory and the organization. Acad. Manag. Rev. 14, 20–39. doi: 10.2307/258189, PMID: 36532993

[ref17] Awino L. O. (2020). The Influence of Corporate Culture on Operational Performance of Multi-National Companies in Kenya. Strathmore University Library.

[ref18] Azeem M. Ahmed M. Haider S. Sajjad M. (2021). Expanding competitive advantage through organizational culture, knowledge sharing and organizational innovation. Technol. Soc. 66:101635. doi: 10.1016/j.techsoc.2021.101635, PMID: 9856002

[ref19] Bachrach D. G. Wang H. Bendoly E. Zhang S. (2007). Importance of organizational citizenship behaviour for overall performance evaluation: comparing the role of task interdependence in China and the USA. Manag. Organ. Rev. 3, 255–276. doi: 10.1111/j.1740-8784.2007.00071.x

[ref20] Bahta D. Yun J. Islam M. R. Bikanyi K. J. (2021). How does CSR enhance the financial performance of SMEs? The mediating role of firm reputation. Econ. Res. 34, 1428–1451. doi: 10.1080/1331677X.2020.1828130

[ref21] Balaji M. Jiang Y. Singh G. Jha S. (2020). Letting go or getting back: how organization culture shapes frontline employee response to customer incivility. J. Bus. Res. 111, 1–11. doi: 10.1016/j.jbusres.2020.02.007, PMID: 36535230

[ref22] Baron R. M. Kenny D. A. (1986). The moderator–mediator variable distinction in social psychological research: conceptual, strategic, and statistical considerations. J. Pers. Soc. Psychol. 51, 1173–1182. doi: 10.1037/0022-3514.51.6.1173, PMID: 3806354

[ref23] Bhoki H. (2020). “The influence of leader member exchange, organizational culture and ethical values on organizational citizenship behavior teacher state senior high Schools in East Flores District” in International Conference on Science and Education and Technology (ISET 2019) (Atlantis Press), 429–435.

[ref24] Bies R. J. (1989). Organizational citizenship behavior: the good soldier syndrome. JSTOR 14:294. doi: 10.2307/258426

[ref25] Blau P.M. (1964). Social Exchange Theory.

[ref26] Cameron K. S. Quinn R. E. (2011). Diagnosing and Changing Organizational Culture: Based on the Competing Values Framework. John Wiley & Sons.

[ref27] Cappelli P. Conyon M. Almeda D. (2020). Social exchange and the effects of employee stock options. ILR Rev. 73, 124–152. doi: 10.1177/0019793919827934, PMID: 36303891

[ref28] Celiker N. Guzeller C. O. (2022). Predicting organizational citizenship behaviour in hospitality businesses with decision tree method. Int. J. Hosp. Tour. Adm., 1–39. doi: 10.1080/15256480.2022.2120942

[ref29] Chatzopoulou E.-C. Manolopoulos D. Agapitou V. (2021). Corporate social responsibility and employee outcomes: interrelations of external and internal orientations with job satisfaction and organizational commitment. J. Bus. Ethics, 1–23. doi: 10.1007/s10551-021-04872-7

[ref30] Chen C.-H. (2022). The mediating effect of corporate culture on the relationship between business model innovation and corporate social responsibility: a perspective from small-and medium-sized enterprises. Asia Pacific. Manag. Rev. 27, 312–319. doi: 10.1016/j.apmrv.2022.01.001

[ref31] Chen Z. Huang S. Liu C. Min M. Zhou L. (2018). Fit between organizational culture and innovation strategy: implications for innovation performance. Sustainability 10:3378. doi: 10.3390/su10103378, PMID: 27885969

[ref32] Cho S. J. Chung C. Y. Young J. (2019). Study on the relationship between CSR and financial performance. Sustainability 11:343. doi: 10.3390/su11020343

[ref33] Dana L.-P. Salamzadeh A. Hadizadeh M. Heydari G. Shamsoddin S. (2022). Urban entrepreneurship and sustainable businesses in smart cities: exploring the role of digital technologies. Sustain. Technol. Entrep. 1:100016. doi: 10.1016/j.stae.2022.100016

[ref34] Del Brio J. Bolaños E. L. (2020). Effects of CSR and CR on business confidence in an emerging country. Sustainability 12:5221. doi: 10.3390/su12125221

[ref35] Dimitrova Y. (2018). The culture of innovation model. Икономически изследвания 27, 39–68.

[ref36] Dmytriyev S. D. Freeman R. E. Hörisch J. (2021). The relationship between stakeholder theory and corporate social responsibility: differences, similarities, and implications for social issues in management. J. Manag. Stud. 58, 1441–1470. doi: 10.1111/joms.12684

[ref37] El-Bassiouny D. El-Bassiouny N. (2018). Diversity, corporate governance and CSR reporting: a comparative analysis between top-listed firms in Egypt, Germany and the USA. Manag. Environ. Qual. Int. J. 30, 116–136. doi: 10.1108/MEQ-12-2017-0150

[ref38] El-Kassar A.-N. Yunis M. Alsagheer A. Tarhini A. Ishizaka A. (2021). Effect of corporate ethics and social responsibility on OCB: the role of employee identification and perceived CSR significance. Int. Stud. Manag. Organ. 51, 218–236. doi: 10.1080/00208825.2021.1959880

[ref39] Ellemers N. Van Der Toorn J. Paunov Y. Van Leeuwen T. (2019). The psychology of morality: a review and analysis of empirical studies published from 1940 through 2017. Personal. Soc. Psychol. Rev. 23, 332–366. doi: 10.1177/1088868318811759, PMID: 30658545 PMC6791030

[ref40] Farhan M. Hussain R. I. Khan S. N. Tahir M. S. Bhatti H. (2020). The relationship among the corporate reputation, customer satisfaction, customer loyalty and behavioral intentions. A study on the Pakistan textile industry. Int. J. Disaster Recov. Bus. Contin. 3:13.

[ref41] Farid T. Iqbal S. Ma J. Castro-González S. Khattak A. Khan M. K. (2019). Employees’ perceptions of CSR, work engagement, and organizational citizenship behavior: the mediating effects of organizational justice. Int. J. Environ. Res. Public Health 16:1731. doi: 10.3390/ijerph16101731, PMID: 31100872 PMC6571754

[ref42] Farmaki A. Pappas N. Kvasova O. Stergiou D. P. (2022). Hotel CSR and job satisfaction: a chaordic perspective. Tour. Manag. 91:104526. doi: 10.1016/j.tourman.2022.104526

[ref43] Farooq M. Farooq O. Cheffi W. (2019). How do employees respond to the CSR initiatives of their organizations: empirical evidence from developing countries. Sustainability 11:2646. doi: 10.3390/su11092646

[ref44] Fornell C. Larcker D. F. (1981). Structural Equation Models with Unobservable Variables and Measurement Error: Algebra and Statistics. Sage Publications Sage CA: Los Angeles, CA, 18, 382.

[ref45] Fu B. Peng J. Wang T. (2022). The health cost of organizational citizenship behavior: does health-promoting leadership matter? Int. J. Environ. Res. Public Health 19:6343. doi: 10.3390/ijerph19106343, PMID: 35627879 PMC9140745

[ref46] González-Rodríguez M. R. Martín-Samper R. C. Köseoglu M. A. Okumus F. (2019). Hotels’ corporate social responsibility practices, organizational culture, firm reputation, and performance. J. Sustain. Tour. 27, 398–419. doi: 10.1080/09669582.2019.1585441

[ref47] Habeeb S. (2019). A proposed instrument for assessing organizational citizenship behavior in BFSI companies in India. Cogent Bus. Manag. 6:1625702. doi: 10.1080/23311975.2019.1625702

[ref48] Hair J. F. Black W. C. Babin B. J. Anderson R. E. Tatham R. L. (1998). Multivariate Data Analysis. Uppersaddle River. Multivariate Data Analysis, vol. 5. 5th Edn Upper Saddle River, 207–219.

[ref49] Hair E. Halle T. Terry-Humen E. Lavelle B. Calkins J. (2006). Children's school readiness in the ECLS-K: predictions to academic, health, and social outcomes in first grade. Early Child. Res. Q. 21, 431–454. doi: 10.1016/j.ecresq.2006.09.005

[ref50] Hamzah M. I. Othman A. K. Fikry A. Abdullah M. Z. (2022). The interaction effects of adhocracy culture, work experience on information acquisition and job performance of bank salespeople. J. Financ. Serv. Mark., 1–14. doi: 10.1057/s41264-022-00166-9

[ref51] Hartnell C. A. Ou A. Y. Kinicki A. J. Choi D. Karam E. P. (2019). A meta-analytic test of organizational culture’s association with elements of an organization’s system and its relative predictive validity on organizational outcomes. J. Appl. Psychol. 104, 832–850. doi: 10.1037/apl0000380, PMID: 30628804

[ref52] Harun M. S. Hussainey K. Kharuddin K. A. M. Al Farooque O. (2020). CSR disclosure, corporate governance and firm value: a study on GCC Islamic banks. Int. J. Account. Inf. Manag. 28, 607–638. doi: 10.1108/IJAIM-08-2019-0103

[ref53] Hassan W. (2020). The effect of entrepreneurial orientation and organisational culture on firm performance: The mediating role of innovation.

[ref54] Hatch M. J. Schultz M. (1997). Relations between organizational culture, identity and image. European. J. Mark.

[ref55] He J. Zhang H. Morrison A. M. (2019). The impacts of corporate social responsibility on organization citizenship behavior and task performance in hospitality: a sequential mediation model. Int. J. Contemp. Hosp. Manag. 31, 2582–2598. doi: 10.1108/IJCHM-05-2018-0378

[ref56] Hou T. C. T. (2019). The relationship between corporate social responsibility and sustainable financial performance: firm-level evidence from Taiwan. Corp. Soc. Responsib. Environ. Manag. 26, 19–28. doi: 10.1002/csr.1647

[ref57] Hu L. T. Bentler P. M. (1999). Cutoff criteria for fit indexes in covariance structure analysis: conventional criteria versus new alternatives. Struct. Equ. Model. Multidiscip. J. 6, 1–55. doi: 10.1080/10705519909540118

[ref58] Huang L. Ma M. Wang X. (2022). Clan culture and risk-taking of Chinese enterprises. China Econ. Rev. 72:101763. doi: 10.1016/j.chieco.2022.101763

[ref59] Ikram M. Sroufe R. Mohsin M. Solangi Y. A. Shah S. Z. A. Shahzad F. (2019). Does CSR influence firm performance? A longitudinal study of SME sectors of Pakistan. J. Glob. Responsibility 11, 27–53. doi: 10.1108/JGR-12-2018-0088

[ref60] Iqbal S. Farid T. Ma J. Khattak A. Nurunnabi M. (2018). The impact of authentic leadership on organizational citizenship behaviours and the mediating role of corporate social responsibility in the banking sector of Pakistan. Sustainability 10:2170. doi: 10.3390/su10072170

[ref61] Isensee C. Teuteberg F. Griese K.-M. Topi C. (2020). The relationship between organizational culture, sustainability, and digitalization in SMEs: a systematic review. J. Clean. Prod. 275:122944. doi: 10.1016/j.jclepro.2020.122944

[ref62] Islam T. Islam R. Pitafi A. H. Xiaobei L. Rehmani M. Irfan M. (2021). The impact of corporate social responsibility on customer loyalty: the mediating role of corporate reputation, customer satisfaction, and trust. Sustain. Prod. Consumption 25, 123–135. doi: 10.1016/j.spc.2020.07.019

[ref63] Jacobsen A. Beehr T. A. (2022). Employees’ death awareness and organizational citizenship behavior: a moderated mediation model. J. Bus. Psychol. 37, 775–795. doi: 10.1007/s10869-021-09772-1, PMID: 34876780 PMC8639849

[ref64] Jeffrey S. Rosenberg S. Mccabe B. (2018). Corporate social responsibility behaviors and corporate reputation. Soc. Responsibility J. 15, 395–408. doi: 10.1108/SRJ-11-2017-0255

[ref65] Jeong Y. Kim E. Kim M. Zhang J. J. (2019). Exploring relationships among organizational culture, empowerment, and organizational citizenship behavior in the south Korean professional sport industry. Sustainability 11:5412. doi: 10.3390/su11195412

[ref66] Kapelus P. (2002). Mining, corporate social responsibility and the" community": the case of Rio Tinto, Richards Bay minerals and the Mbonambi. J. Bus. Ethics 39, 275–296. doi: 10.1023/A:1016570929359

[ref67] Karyawati G. Subroto B. Sutrisno T. Saraswati E. (2020). Explaining the complexity relationship of CSR and financial performance using neo-institutional theory. J. Asian Bus. Econ. Stud. doi: 10.1108/JABES-10-2019-0106

[ref68] Kawiana I. G. P. Dewi L. K. C. Martini L. K. B. Suardana I. B. R. (2018). The influence of organizational culture, employee satisfaction, personality, and organizational commitment towards employee performance. Int. Res. J. Manag. IT Soc. Sci. 5, 35–45.

[ref69] Khan W. Hassan R. Arshad M. Arshad M. Kashif U. Aslam F. (2020). The effect of entrepreneurial orientation and organisational culture on firm performance: the mediating role of innovation. Int. J. Innov. Creat. Change 13, 652–677.

[ref70] Khan R. U. Salamzadeh Y. Iqbal Q. Yang S. (2022). The impact of customer relationship management and company reputation on customer loyalty: the mediating role of customer satisfaction. J. Relat. Mark. 21, 1–26. doi: 10.1080/15332667.2020.1840904

[ref71] Khaskheli A. Jiang Y. Raza S. A. Qureshi M. A. Khan K. A. Salam J. (2020). Do CSR activities increase organizational citizenship behavior among employees? Mediating role of affective commitment and job satisfaction. Corp. Soc. Responsib. Environ. Manag. 27, 2941–2955. doi: 10.1002/csr.2013

[ref72] Kheir-Faddul N. Bibu N. Nastase M. (2019). The PRINCIPALS'PERCEPTION of their values and the organizational culture of the junior high SCHOOLS in the DRUZE sector. Rev. Manag. Comparat. Int. 20, 210–225.

[ref73] Kim M. Yin X. Lee G. (2020). The effect of CSR on corporate image, customer citizenship behaviors, and customers’ long-term relationship orientation. Int. J. Hosp. Manag. 88:102520. doi: 10.1016/j.ijhm.2020.102520

[ref74] Kozhakhmet S. Nurgabdeshov A. (2022). Knowledge acquisition of Chinese expatriates: managing Chinese MNEs in Kazakhstan. J. Int. Manag. 28:100919. doi: 10.1016/j.intman.2021.100919

[ref75] Kudłak R. Szőcs I. Krumay B. Martinuzzi A. (2018). The future of CSR-selected findings from a Europe-wide Delphi study. J. Clean. Prod. 183, 282–291. doi: 10.1016/j.jclepro.2018.02.119

[ref76] Lai C.-S. Chiu C.-J. Yang C.-F. Pai D.-C. (2010). The effects of corporate social responsibility on brand performance: the mediating effect of industrial brand equity and corporate reputation. J. Bus. Ethics 95, 457–469. doi: 10.1007/s10551-010-0433-1

[ref77] Le T. T. (2022). Corporate social responsibility and SMEs' performance: mediating role of corporate image, corporate reputation and customer loyalty. Int. J. Emerg. Mark. doi: 10.1108/IJOEM-07-2021-1164

[ref78] Lee Y. (2021). Linking internal CSR with the positive communicative behaviors of employees: the role of social exchange relationships and employee engagement. Soc. Respons. J. 18, 348–367. doi: 10.1108/SRJ-04-2020-0121

[ref79] Lee Y. Tao W. (2020). Employees as information influencers of organization’s CSR practices: the impacts of employee words on public perceptions of CSR. Public Relat. Rev. 46:101887. doi: 10.1016/j.pubrev.2020.101887

[ref80] Li J. Fung H. (2020). Culture at work: European American and Taiwanese parental socialization of children’s learning. Appl. Dev. Sci. 25, 26–37. doi: 10.1080/10888691.2020.1789351

[ref81] Li H. Liu H. Zhao H. (2021). Traditional culture echoes? The impact of clan culture upon partner surname sharing: evidence from Chinese supply chains. Ind. Mark. Manag. 99, 40–53. doi: 10.1016/j.indmarman.2021.09.008

[ref82] Lockhart P. Shahani N. K. Bhanugopan R. (2020). Do organisational culture and national culture mediate the relationship between high-performance human resource management practices and organisational citizenship behaviour? Int. J. Manpow. 41, 1179–1197. doi: 10.1108/IJM-04-2018-0129

[ref83] Mansouri A. A. A. Singh S. K. Khan M. (2018). Role of organisational culture, leadership and organisational citizenship behaviour on knowledge management. Int. J. Knowl. Manag. Stud. 9, 129–143. doi: 10.1504/IJKMS.2018.091249, PMID: 29923649

[ref84] Manzoor F. Wei L. Asif M. (2021). Intrinsic rewards and employee's performance with the mediating mechanism of employee's motivation. Front. Psychol. 12:563070. doi: 10.3389/fpsyg.2021.563070, PMID: 34335346 PMC8319625

[ref85] Manzoor F. Wei L. Bányai T. Nurunnabi M. Subhan Q. A. (2019a). An examination of sustainable HRM practices on job performance: an application of training as a moderator. Sustainability 11:2263. doi: 10.3390/su11082263

[ref86] Manzoor F. Wei L. Nurunnabi M. Subhan Q. A. Shah S. I. A. Fallatah S. (2019b). The impact of transformational leadership on job performance and CSR as mediator in SMEs. Sustainability 11:436. doi: 10.3390/su11020436

[ref87] Martínez P. Pérez A. Rodríguez Del Bosque I. (2014). Exploring the role of CSR in the organizational identity of hospitality companies: a case from the Spanish tourism industry. J. Bus. Ethics 124, 47–66. doi: 10.1007/s10551-013-1857-1

[ref88] Masood S. A. Dani S. S. Burns N. D. Backhouse C. J. (2006). Transformational leadership and organizational culture: the situational strength perspective. Proc. Inst. Mech. Eng. B J. Eng. Manuf. 220, 941–949. doi: 10.1243/09544054JEM499

[ref89] Michaelis B. Stegmaier R. Sonntag K. (2009). Affective commitment to change and innovation implementation behavior: the role of charismatic leadership and employees’ trust in top management. J. Chang. Manag. 9, 399–417. doi: 10.1080/14697010903360608

[ref90] Mikołajczak P. (2020). Social enterprises’ hybridity in the concept of institutional logics: evidence from polish NGOs. Volunt. Int. J. Volunt. Nonprofit Org. 31, 472–483. doi: 10.1007/s11266-020-00195-9

[ref91] Misigo G. K. Were S. Odhiambo R. (2019). Influence of adhocracy culture on performance of public water companies in Kenya. Int. Acad. J. Hum. Resour. Bus. Adm. 3, 84–103.

[ref92] Mitonga-Monga J. (2019). Examining organisational citizenship behaviour as an outcome of an ethical work culture in a developing country. J. Contemp. Manag. 16, 333–356. doi: 10.35683/jcm18057.0017

[ref93] Mitonga-Monga J. Flotman A.-P. (2021). Corporate ethical values and ethical work climate influences on employees’ helping behaviour in a developing country’s banking sector setting. J. Psychol. Afr. 31, 580–587. doi: 10.1080/14330237.2021.2001920

[ref94] Nanayakkara K. Wilkinson S. (2021). “Organisational culture theories: dimensions of organisational culture and office layouts” in A Handbook of Theories on Designing Alignment Between People and the Office Environment (Routledge)

[ref95] Naz S. Li C. Nisar Q. A. Khan M. A. S. Ahmad N. Anwar F. (2020). A study in the relationship between supportive work environment and employee retention: role of organizational commitment and person–organization fit as mediators. SAGE Open 10:215824402092469. doi: 10.1177/2158244020924694

[ref96] Nguyen N. T. T. Nguyen N. P. Hoai T. T. (2021). Ethical leadership, corporate social responsibility, firm reputation, and firm performance: a serial mediation model. Heliyon 7:e06809. doi: 10.1016/j.heliyon.2021.e06809, PMID: 33898855 PMC8060600

[ref97] Nill A. Papp B. L. (2020). “CSR in the USA: a historic perspective on the interplay between ideological, political, and economic forces,” in Rethinking Business Responsibility in a Global Context (Springer).

[ref98] Noone B. M. Lin M. S. Sharma A. (2022). Firm performance during a crisis: effects of adhocracy culture, incremental product innovation, and firm size. J. Hosp. Tour. Res. doi: 10.1177/10963480221086

[ref99] Oliveira T. Thomas M. Baptista G. Campos F. (2016). Mobile payment: understanding the determinants of customer adoption and intention to recommend the technology. Comput. Hum. Behav. 61, 404–414. doi: 10.1016/j.chb.2016.03.030

[ref100] Oo E. Y. Jung H. Park I.-J. (2018). Psychological factors linking perceived CSR to OCB: the role of organizational pride, collectivism, and person–organization fit. Sustainability 10:2481. doi: 10.3390/su10072481

[ref101] Osakwe C. N. Ruiz B. Amegbe H. Chinje N. B. Cheah J.-H. Ramayah T. (2020). A multi-country study of bank reputation among customers in Africa: key antecedents and consequences. J. Retail. Consum. Serv. 56:102182. doi: 10.1016/j.jretconser.2020.102182

[ref102] Özcan F. Elçi M. (2020). Employees’ perception of CSR affecting employer brand, brand image, and corporate reputation. SAGE Open 10:2158244020972372. doi: 10.1177/2158244020972372

[ref103] Park E. (2019). Corporate social responsibility as a determinant of corporate reputation in the airline industry. J. Retail. Consum. Serv. 47, 215–221. doi: 10.1016/j.jretconser.2018.11.013

[ref104] Pasricha P. Singh B. Verma P. (2018). Ethical leadership, organic organizational cultures and corporate social responsibility: an empirical study in social enterprises. J. Bus. Ethics 151, 941–958. doi: 10.1007/s10551-017-3568-5

[ref105] Pires V. Trez G. (2018). Corporate reputation: a discussion on construct definition and measurement and its relation to performance. Rev. Gestão. 25, 47–64. doi: 10.1108/REGE-11-2017-005

[ref106] Podsakoff P. M. Organ D. W. (1986). Self-reports in organizational research: problems and prospects. J. Manag. 12, 531–544. doi: 10.1177/014920638601200408, PMID: 33351435

[ref107] Preacher K. J. Hayes A. F. (2008). Asymptotic and resampling strategies for assessing and comparing indirect effects in multiple mediator models. Behav. Res. Methods 40, 879–891. doi: 10.3758/BRM.40.3.879, PMID: 18697684

[ref108] Rabiul M. K. Yean T. F. Patwary A. K. Mohamed A. E. Hilman H. (2021). Construct validation of leaders’ motivating language in the context of the hotel industries of Bangladesh and Malaysia. Int. J. Contemp. Hosp. Manag. 33, 2695–2720. doi: 10.1108/IJCHM-01-2021-0096

[ref109] Rehman Z. U. Khan A. Rahman A. (2020). Corporate social responsibility's influence on firm risk and firm performance: the mediating role of firm reputation. Corp. Soc. Responsib. Environ. Manag. 27, 2991–3005. doi: 10.1002/csr.2018

[ref110] Rupp D. E. Ganapathi J. Aguilera R. V. Williams C. A. (2006). Employee reactions to corporate social responsibility: an organizational justice framework. J. Organ. Behav. 27, 537–543. doi: 10.1002/job.380

[ref111] Sackmann S. A. (2021). Culture and Organizational Performance. Culture in Organizations. Springer.

[ref112] Sallah C. A. Caesar L. D. (2020). Intangible resources and the growth of women businesses: empirical evidence from an emerging market economy. J. Entrep. Emerg. Econ. 12, 329–355. doi: 10.1108/JEEE-05-2019-0070

[ref113] Scaliza J. A. A. Jugend D. Jabbour C. J. C. Latan H. Armellini F. Twigg D. (2022). Relationships among organizational culture, open innovation, innovative ecosystems, and performance of firms: evidence from an emerging economy context. J. Bus. Res. 140, 264–279. doi: 10.1016/j.jbusres.2021.10.065

[ref114] Schein P.A. (2004). Organizational Culture and Leadership.

[ref115] Shaked H. (2021). How clan culture impairs functions of instructional leadership: the case of Israel. Leadersh. Policy Sch., 1–15. doi: 10.1080/15700763.2021.1966048

[ref116] Shin I. Hur W.-M. Kang S. (2016). Employees’ perceptions of corporate social responsibility and job performance: a sequential mediation model. Sustainability 8:493. doi: 10.3390/su8050493, PMID: 36011990

[ref117] Singh K. Misra M. (2021). Linking corporate social responsibility (CSR) and organizational performance: the moderating effect of corporate reputation. Eur. Res. Manag. Bus. Econ. 27:100139. doi: 10.1016/j.iedeen.2020.100139

[ref118] Soelton M. Visano N. A. Noermijati N. Ramli Y. Syah T. Y. R. Sari Y. J. (2020). The implication of job satisfaction that influence workers to practice organizational citizenship behavior (OCB) in the work place. Arch. Bus. Rev. 8.

[ref119] Stojanovic A. Milosevic I. Arsic S. Urosevic S. Mihajlovic I. (2020). Corporate social responsibility as a determinant of employee loyalty and business performance. J. Compet. 12, 149–166. doi: 10.7441/joc.2020.02.09

[ref120] Supanti D. Butcher K. (2019). Is corporate social responsibility (CSR) participation the pathway to foster meaningful work and helping behavior for millennials? Int. J. Hosp. Manag. 77, 8–18. doi: 10.1016/j.ijhm.2018.06.001

[ref121] Tadelis S. (2003). Firm reputation with hidden information. Economic Theory 21, 635–651. doi: 10.1007/s00199-002-0257-z

[ref122] Tangngisalu J. Mappamiring M. Andayani W. Yusuf M. Putra A. H. P. K. (2020). CSR and firm reputation from employee perspective. J. Asian Finance Econ. Bus. 7, 171–182. doi: 10.13106/jafeb.2020.vol7.no10.171

[ref123] Tran T. B. H. Choi S. B. (2019). Effects of inclusive leadership on organizational citizenship behavior: the mediating roles of organizational justice and learning culture. Journal of Pacific rim. Psychology 13. doi: 10.1017/prp.2019.10

[ref124] Verčič A. T. Ćorić D. S. (2018). The relationship between reputation, employer branding and corporate social responsibility. Public Relat. Rev. 44, 444–452. doi: 10.1016/j.pubrev.2018.06.005, PMID: 22699256

[ref125] Waheed A. Zhang Q. (2020). Effect of CSR and ethical practices on sustainable competitive performance: a case of emerging markets from stakeholder theory perspective. J. Bus. Ethics. 175, 1–19. doi: 10.1007/s10551-020-04679-y

[ref126] Wang W. Fu Y. Qiu H. Moore J. H. Wang Z. (2017). Corporate social responsibility and employee outcomes: a moderated mediation model of organizational identification and moral identity. Front. Psychol. 8:1906. doi: 10.3389/fpsyg.2017.01906, PMID: 29163287 PMC5671997

[ref127] Wang X.-H. Yang J. Cao R. Lee B. Y. (2019). Corporate social responsibility and collective OCB: a social identification perspective. Front. Psychol. 10:2720. doi: 10.3389/fpsyg.2019.02720, PMID: 31920789 PMC6917590

[ref128] Wang C. Zhang Q. Zhang W. (2020). Corporate social responsibility, green supply chain management and firm performance: the moderating role of big-data analytics capability. Res. Transp. Bus. Manag. 37:100557. doi: 10.1016/j.rtbm.2020.100557

[ref129] Wiedmann K.-P. Hennigs N. Schmidt S. Wuestefeld T. (2013). Brand heritage and its impact on corporate reputation: corporate roots as a vision for the future. Corp. Reput. Rev. 16, 187–205. doi: 10.1057/crr.2013.10

[ref130] Wisse B. Van Eijbergen R. Rietzschel E. F. Scheibe S. (2018). Catering to the needs of an aging workforce: the role of employee age in the relationship between corporate social responsibility and employee satisfaction. J. Bus. Ethics 147, 875–888. doi: 10.1007/s10551-015-2983-8

[ref131] Xie Y. Gu D. Liang C. Zhao S. Ma Y. (2020). How transformational leadership and clan culture influence nursing staff's willingness to stay. J. Nurs. Manag. 28, 1515–1524. doi: 10.1111/jonm.13092, PMID: 32656804

[ref132] Yaakobi E. Weisberg J. (2020). Organizational citizenship behavior predicts quality, creativity, and efficiency performance: the roles of occupational and collective efficacies. Front. Psychol. 11:758. doi: 10.3389/fpsyg.2020.00758, PMID: 32390915 PMC7193106

[ref133] Yadav R. S. Dash S. S. Chakraborty S. Kumar M. (2018). Perceived CSR and corporate reputation: the mediating role of employee trust. Vikalpa 43, 139–151. doi: 10.1177/0256090918794823

[ref134] Yan X. Espinosa-Cristia J. F. Kumari K. Cioca L. I. (2022). Relationship between corporate social responsibility, organizational trust, and corporate reputation for sustainable performance. Sustainability 14:8737. doi: 10.3390/su14148737, PMID: 34777154

[ref135] Yang T. Jiang X. Cheng H. (2022). Employee recognition, task performance, and OCB: mediated and moderated by pride. Sustainability 14:1631. doi: 10.3390/su14031631

[ref136] Yoon B. Chung Y. (2018). The effects of corporate social responsibility on firm performance: a stakeholder approach. J. Hosp. Tour. Manag. 37, 89–96. doi: 10.1016/j.jhtm.2018.10.005, PMID: 34071620

[ref137] Youn H. Kim J.-H. (2022). Corporate social responsibility and hotel employees’ organizational citizenship behavior: the roles of organizational pride and meaningfulness of work. Sustainability 14:2428. doi: 10.3390/su14042428

[ref138] Zeb A. Akbar F. Hussain K. Safi A. Rabnawaz M. Zeb F. (2021). The competing value framework model of organizational culture, innovation and performance. Bus. Process. Manag. J. 27, 658–683. doi: 10.1108/BPMJ-11-2019-0464, PMID: 11989961

[ref139] Zeyada M. (2018). Organizational culture and its impact on organizational citizenship behavior. Int. J. Acad. Res. Bus. Soc. Sci. 8, 418–429.

[ref140] Zhang Q. Ahmad S. (2021). Analysis of corporate social responsibility execution effects on purchase intention with the moderating role of customer awareness. Sustainability 13:4548. doi: 10.3390/su13084548

[ref141] Zhang Y. Tsang K. K. Wang L. Liu D. (2022). Emotional labor mediates the relationship between clan culture and teacher burnout: an examination on gender difference. Sustainability 14:2260. doi: 10.3390/su14042260

[ref142] Ziaei Nafchi M. Mohelská H. (2020). Organizational culture as an indication of readiness to implement industry 4.0. Information 11:174. doi: 10.3390/info11030174

[ref143] Žukauskas P. (2018). Organizational Culture and Leadership.

